# Indents

**Published:** 1921-03

**Authors:** 


### INDENTS

**☞Brief Articles of Technical or Scientific Value will receive notice and comment. Contributions invited.**

---

Defective Teeth Among School Children. The condition of the teeth of school children has received considerable attention during the last few years and various attempts have been made to obtain statistics on unhealthy teeth. A report published by Dr. Royal S. Copland, Health Commissioner of New York City, contains some facts relative to school children that can hardly be passed without comment. Dr. Copland has made a comparison of the physical defects of school children based on conditions as found in 1909 and 1919. The report as published in the *New York Times* does not state the number of children examined or in what part of the city the examinations were made, but we presume it was a composite report covering examinations made over the entire city of New York. Not only were the teeth examined, but other physical defects were noted. A comparison of these defects as found in 1909 and 1919 is quite interesting.

The report covers ten physical defects, seven of which show a decrease over the ten years between 1909 and 1919. The only three that show an increase are malnutrition, cardiac defects, and defective teeth. Beginning with defective vision, we notice a 6 per cent. reduction in the two tabulations. Defective hearing in 1909 was 1.0 per cent.; in 1919, 0.5 per cent., which would be half as many with defective hearing in 1919 as were found in 1909. Defective nasal breathing also fell from 18.1 per cent, to 11.6 per cent.; hypertrophied tonsils, from 22.0 per cent. to 15.3 per cent. When we come to malnutrition we notice in 1909, 3.1 per cent. in 1919, 19.9 per cent. This means that where one child was found suffering from malnutrition in 1909, six were found in 1919, or an increase of 600 per cent. in ten years. This ratio is entirely too great, and we can find no reason for it. We wonder whether some of the difference is not the result of a more careful examination made at the present time than was made in 1909. It has only been comparatively few years that the medical profession has made the careful examination for malnutrition that it now made, and it is very probable that an increase in number is the result of improved examination methods. Cardiac disease also shows an increase from 0.65 per cent. in 1909 to 1.5 per cent in 1919. This increase should be more if malnutrition actually increased as much as the report shows. Pulmonary diseases show a slight decrease over the ten-year period, which also is strange if malnutrition has increased, for it is a well-known fact that pulmonary diseases always increase as malnutrition becomes greater.

When we come to defective teeth, we find 57.0 per cent. in 1909 and 62.3 per cent. in 1919. This increase in ten years we think is also the result of a more careful examination than was made in 1909. If there has been an actual increase in malnutrition, we would expect to note an increase in defective teeth. However, in the face of other conditions as tabulated, we are inclined to doubt an actual increase as extending over a period of ten years. Until there are other reports turned in, we are inclined to believe the increase of dental defects from 1909 to 1919 has been the result of more careful examinations and not an increase in pathologic conditions.—*International Journal of Orthodontia.*

---

THE CLINICAL ASPECTS OF THE DENTAL SITUATION. An incident which came recently under our observation leads us once again to undertake the task of pointing out certain grave dangers which are always associated with surgical operations in the mouth. The similarity in the effects of vaccines when injected into the body fluids for curative or prophylactic purposes and of operations in the mouth entailing the tearing of soft tissue has been repeatedly pointed out in these pages. It is a difference in degree, but not in kind. In the former case the quantity of vaccine to be administered is determinable and is decided upon on the basis of generally reliable indications, viz., differential blood count, hemoglobin percentage, blood pressure, temperature range, the existence of visceral lesions, etc. In the latter case—operations about the mouth—they are performed ordinarily with disregard of these data. It is an anomalous situation that which is revealed by the attitude of the dental world. We find everywhere in dental journals allusions to the need of greater familiarity with pathology, general and dental, bacteriology, immunology, etc., but so far we have failed to find a generalized individual desire to put these doctrines into practice. A group of dental investigators has been guiding the way to a better understanding of our problems. They have assimilated the results of the labors of those scientists to whom we owe so much for their valuable contributions to dentistry in the past thirty years and together with their own findings are helping to clarify the dental situation. We should be able to find a reflection of these unselfish endeavors in the quality of the reparative and preventive operations in the mouth. All the labor and time which has been devoted in the past for the improvement of dental interventions and which is being devoted today by investigators in the various departments of dentistry has not borne, so far as can be detected in the mouth, the hoped-for fruit. There is seemingly a lack of endeavor to put into practice those teachings which because they are the outcome of scientific evidence are the safe ones to follow from the standpoint of the patient's future welfare. Less individual dogmatizing and a greater effort toward the attainment of the ideal, less reiteration of well-known facts and more concentration upon the clinical application of sound principles, less speculative conclusions and a closer adherence to facts as discovered in the clinic or the laboratory—these we hope may be the planks in the dental platform of the future.

Reverting to our incident. It concerned a patient who had been admonished to have but one tooth extracted at a sitting and to wait three days before other extractions were performed. By the operator to whom the patient was referred this admonition seemed an unwarranted overcautiousness. The tooth was extracted in the afternoon and in the evening of the same day the patient was overcome by the auto-vaccination. Chills and a temperature of 103° F. plus physical and mental depression were the symptoms. What would have been the termination of this case, if the three incurable teeth had been removed at once, can readily be surmised. Symptoms of the same order, though less severe, are prone to follow extensive gingival traumatism in the course of treatments for pyorrhea alveolaris and gingivitis and the development of infections in the respiratory passages following dental operations has been noted. The information necessary for the comprehension of these problems is available to every dental practitioner. It has been at his elbow-reach for many years—although it is possible that the magazines in which it is to be found are still in their original covers. —*Pacific Dental Journal.*

---

"DEAD" TEETH AND "DEVITALIZED" TEETH. Modern life insurance companies are keenly interested in all movements calculated to improve the health and prolong the lives of the people. The larger insurance companies are now spending large sums of money annually in health propaganda among their policy holders and the general public. All such efforts are to be commended. Insurance companies who undertake educational work should, however, fully assure themselves that the literature they publish upon health subjects is in accord with the scientific thought and best judgment of recognized practitioners.

One of the largest companies publishes a quarterly magazine, known as *The Imperial Life-Guard.* In the *Life-Guard* for December, 1920, the subject of ''Bad Teeth'' is discussed under the general heading, ''How to Keep Well,'' and some of the statements appearing therein will be somewhat in the nature of a surprise to the Dental Profession. The following quotation speaks for itself:

''Having the teeth 'fixed up' by killing the nerves, filling and sealing up the cavities, means that future trouble will likely arise. * * * A mouthful of gold is a menace. * * * A devitalized tooth is a dead tooth and is likely to act just the same as any other foreign body embedded in the tissues. If a tooth can not be filled or otherwise treated without killing the nerve, it should be extracted.

''How many hard-working business men with beautifully gold-decorated, devitalized teeth, have noticed their usual energy decreasing—a feeling of getting old prematurely—and wondered what the cause could be? How often have they regained their old-time vim and feeling of well-being soon after having old, dead, root infected filled teeth removed? Again, how often have they become cases for the physician or surgeon because the trouble progressed unrecognized?

''Ask your modern, up-to-date dentist his opinion regarding the dangers from dead mechanically sealed up teeth. With luck such teeth may cause no trouble for years, but the odds are a long way against this.

''*Safe-Guards.* 1. Visit a good dentist twice a year. 2. Have those dead teeth X-rayed once a year. Serious root trouble, which often is not apparent outwardly, can be brought to light by the X-ray. 3. If X-ray plate shows evidence of disease, take it at once to your dentist and have the root-infected teeth extracted. 4. Remember that the infection is easily carried from teeth to vital organs of the body.''

Many statements in the above article are out of harmony with the best thought of the dental profession, that to the ''modern, up-to-date dentist'' they sound positively ridiculous.

The dental profession fully realizes the need for public instruction in the prevention of dental disease and the care of the teeth, and stands ready to co-operate in every possible way with other agencies working along similar lines. But surely publishers might be expected to first submit manuscript of this sort to the official committees of the dental profession, which are specially appointed to deal with matters of this kind.

It is unfortunate that any ''health'' publication should associate without question ''a mouthful of gold'' with dental disease, as though the ''gold'' and the ''disease'' were related. And to then refer to ''devitalized'' teeth as being ''dead'' teeth. The advice in Safe-Guard No. 2 to ''have those 'dead' teeth X-rayed once a year'' is made ridiculous through the writer's apparent misconception that the words *dead* and *devitalized* are synonymous terms, and Safe-Guard No. 3 apparently presupposes that the dental X-ray is taken by someone other than a dentist, a diagnosis made, and then (when the treatment has been determined), ''take it at once to your dentist and have the root-infected teeth extracted.'' Apparently the author of the article is worthy to be elected an honorary member of the socalled one hundred per cent. club. Many people who feel free to thoughtlessly advise their friends to have their teeth extracted, are much more considerate of their own teeth. Thousands upon thousands of devitalized teeth have been saved in health by dental surgeons and continue to render good service to their owners. Dentistry stands for the extraction of all teeth that can not be made healthy, but dentistry also stands for the saving of the tooth, in the interests of health, efficient mastication, and natural appearance, in every case where the tooth can be saved in a condition of health.—*Oral Health* of January.

---

THE QUESTIONARY ON AMALGAM. The following questions were sent out to the profession some months ago by the Caulk Research Department:

(1) In cases of pitted or corroded amalgam fillings that have come under your personal observation, do you know what alloys were used—high or low silver? zinc or non-zinc? (2) Was the amalgam in direct contact with a gold crown or filling or could it come in contact with the gold on closing the mouth? (3) Can you state whether the gold or the amalgam, or both, were insulated from the tooth by cement or other material? (4) Were the cavities deep-seated or shallow? (5) In your judgment, were the fillings properly made? (6) Under what conditions have you observed galvanic shock or injury to the pulp? (7) What evidence have you regarding deterioration of dental alloys or amalgams due to age? (8) What, in your judgment, are the essential points in amalgam technique, and why? (9) Can you suggest any questions on alloys or amalgams which, as far as you know, have never been answered? (10) Shall we send you, free of charge, a copy of our forthcoming book?

About a thousand replies were received within a week after the letter was mailed. And the total number has not yet been reached. The work of analyzing and co-ordinating these thousands of opinions has gone steadily forward in connection with the preparation of the Caulk book on amalgams. This book will be published just as soon as the research work, which is now going on, can be completed and described. Additional answers to the questions, and any other information on the subject, will be appreciated. —*Dental Quarterly*.

---

PORCELAIN PONTICS. A porcelain pontic is not necessarily a porcelain root. It is well to be clear on this point, as there has been some confusion in the use of the terms, they having been used synonymously on occasions. A pontic may touch the gum tissue or it may not. The writer has no desire to laud the porcelain root and believes it should be seldom used, but will defend the porcelain pontic from every standpoint.

We use porcelain because of its esthetic and sanitary qualities. Consequently the more surfaces of the pontic that can be covered with porcelain without weakening it the better. There are several different types of porcelain pontics. For bicuspids and molars the pontics are made by baking porcelain on to the ridge-lap of long-pin facings so as to make the gingival and a portion of the linqual surface of porcelain, having a dome shape. It is quite essential to use the long-pin facings, as the short pins are not of sufficient length to hold when cemented to the gold backings.

The gold backing is made by filling in with wax around the pins, having previously oiled the porcelain to prevent the wax from sticking, carving to the desired form on the occlusal and remaining lingual surface; remove and insert carbon points in pin holes; invest and cast.

In the six anterior teeth the same method may be employed by using the long-pin facings and following out the technic as above described. There are some objections to this type of pontic for anterior teeth, owing to the necessity of extending the gold backing down to the incisal edge and also the necessity of using long-pin facings, the length of which frequently interferes with the occlusion of the lower teeth.

To overcome these objections, the writer has designed a type of pontic for the six anterior teeth. This is made by using any pinless porcelain crown as a base and baking on porcelain to the desired form, changing the direction of the pin hole to a certain extent so that the porcelain may be made so that it will fill the space to the gum on the lingual as well as on the labial. This type of pontic fills every requirement from an esthetic standpoint as well as that of durability in most cases.—J. E. Rose, in *N. W. Journal.*

---

To Roughen a Broach. Fold a carborundum paper disk —medium grit—across center and pinch together while drawing broach between folds. If desired to withdraw cotton from canal with broach rotate it backward and forward while drawing through disk. If cotton is to be left in canal with dressing draw through disk without rotation; a slight backward turn will free the broach and leave treatment. Examine prepared broach with magnifying glass and reason will be apparent.—J. T. Search, in *Dental Facts.*

---

To Refit an Upper Vulcanite Plate. Take impression and model same as for any plate. With mechanical saw cut out all the palatal portion of old plate to within a quarter of an inch of the pins. Scarify the palatal part of what is left and thoroughly clean with chloroform. Now mix Dougherty's vulcanizable gutta-percha with chloroform to the consistency of soft putty and spread over the portion of the plate that goes over ridge and press to place on the model. Also paint the model with this paste, but made thin. Fill the palatal portion with the sheet vulcanizable gutta-percha, flask and vulcanize.—E. T. Evans, in *Dental Facts.*

### BETTER EXPRESS SERVICE AIM OF RIGHT WAY PLAN

An effort to improve the express service of the country, is being made by The American Railway Express Company, which has announced the inauguration of a Right Way Plan, under which special attention is to be paid to the correct methods of handling express matter.

Shippers in every industry, which require express transportation in their business, will be asked to aid in the movement by "Starting their shipments right." The carrier announces that having received traffic in proper shipping condition, it proposes to see that the shipments while in its hands will be carefully and expeditiously handled.

"Starting shipments right," according to the announcement of the carrier, involves special attention to full and accurate addressing of shipments and packing, according to the rules laid down in the express classification, authorized by the Interstate Commerce Commission. Correct methods for filing claims, for sending shipments C. O. D. and other essentials of express shipping, will be brought to the attention of the shipping public.

The Right Way Plan also includes an ambitious educational program to be carried on throughout the express ranks, covering all the important points of proper express methods for handling the business, after having been received from the public. The first meeting under the plan will be called on January 11th, when express workers in every city where express matter is handled will be brought together and addressed by officials on the most important topics of the hour in the business.

This educational work is to be carried on largely by the employes themselves, through special Right Way Committees, selected by officials, from the most experienced workers in the various local offices.

